# Agronomic iodine biofortification of leafy vegetables grown in Vertisols, Oxisols and Alfisols

**DOI:** 10.1007/s10653-020-00714-z

**Published:** 2020-09-23

**Authors:** Ivy Sichinga Ligowe, E. H. Bailey, S. D. Young, E. L. Ander, V. Kabambe, A. D. Chilimba, R. M. Lark, P. C. Nalivata

**Affiliations:** 1 Lilongwe University of Agriculture and Natural Resources, Bunda Campus, P.O. Box 219, Lilongwe, Malawi; 2 Department of Agricultural Research Services, P.O. Box 30779, Lilongwe 3, Malawi; 3 School of Biosciences, University of Nottingham, Sutton Bonington Campus, Loughborough, LE12 5RD UK; 4 Centre for Environmental Geochemistry, British Geological Survey, Nottingham, NG12 5GG UK

**Keywords:** Iodine biofortification, *Brassica napus* L*.*, *Amaranthus retroflexus* L., Vertisols, Oxisols, Alfisols

## Abstract

Iodine deficiency disorders (IDD) in sub-Saharan African countries are related to low dietary *I* intake and generally combatted through salt iodisation. Agronomic biofortification of food crops may be an alternative approach. This study assessed the effectiveness of *I* biofortification of green vegetables (*Brassica napus* L and *Amaranthus retroflexus* L.) grown in tropical soils with contrasting chemistry and fertility. Application rates of 0, 5 and 10 kg ha^−1^ *I* applied to foliage or soil were assessed. Leaves were harvested fortnightly for ~ 2 months after *I* application before a second crop was grown to assess the availability of residual soil *I*. A separate experiment was used to investigate storage of *I* within the plants. Iodine concentration and uptake in sequential harvests showed a sharp drop within 28 days of *I* application in all soil types for all *I* application levels and methods. This rapid decline likely reflects *I* fixation in the soil. Iodine biofortification increased *I* uptake and concentration in the vegetables to a level useful for increasing dietary *I* intake and could be a feasible way to reduce IDD in tropical regions. However, biofortification of green vegetables which are subject to multiple harvests requires repeated *I* applications.

**Electronic supplementary material:**

The online version of this article (10.1007/s10653-020-00714-z) contains supplementary material, which is available to authorised users.

## Introduction

Iodine (*I*) is a constituent of thyroid hormones and critical for metabolism and overall human health (Ujowundu et al. 2011). The recommended daily allowance (RDA) of *I* for adults is 150–290 μg d^−1^ with a tolerable upper limit of 1100 μg d^−1^ (Patrick 2008; WHO 2007). Iodine deficiency disorders (IDDs) are widespread in both developed and developing countries, affecting between 800 million and 2 billion people worldwide (Dasgupta et al. 2008; Szybinski et al. 2010). Sources of *I* include fish and dairy products (Pehrsson et al. 2016) with consumption of these food products largely dependent upon geographical location and the socio-economic status of individuals (Joy et al. 2015; Weng et al. 2013). In many countries, salt iodisation has been adopted with the aim of increasing dietary *I* intake to combat risks of IDD. However, the concentration of *I* in salt is highly variable and dependent on level of iodisation at manufacture and subsequent losses in cooking or through improper storage (Kalimbira et al. 2005; Diosady et al. 1998). Losses of up to 20% can occur during production, packaging, transportation and processing, with cooking processes contributing an additional loss of 20% (Winger et al. 2008; WHO 2007). Additionally, as healthy adults are advised to lower their salt intake to reduce risks of cardiovascular disease and kidney damage, it is reported that three quarters may no longer consume sufficient *I* through iodised salt intake (Piccone 2011). The reduction in iodised salt intake therefore may increase the incidence of IDDs. In the developed world, *I* deficiency has increased more than fourfold over the past 40 years (Verduzco-Gallo et al. 2014; Szybinski et al. 2010). In sub-Saharan countries such as Malawi, although salt iodisation has been declared adequate to meet recommended dietary *I* intake (IGN 2017), salt intake varies according to cultural and economic factors (Joy et al. 2015). Supply of *I* from foods other than salt is inadequate to meet the requirements of almost 100% of households in Malawi, and were this not present in the Malawian diet, there would be a typical dietary *I* intake of 12 µg day^−1^ (Joy et al. 2015) and the same is true across much of the world.

One approach to tackling IDD is optimisation of soil and crop management strategies to increase *I* uptake into the edible portions of widely consumed food crops, e.g. through foliar or soil biofortification, an approach which has shown considerable promise for other mineral micronutrients, such as zinc and selenium (Chilimba et al. 2012; Manzeke et al. 2014). Green vegetables are amongst the most common food crops grown and consumed by Malawians, irrespective of origin, race, age, sex or economic status (Gondwe 2008). They contribute greatly to the nutritional well-being of rural people by providing essential nutrients for growth and development and prevention of diseases associated with nutritional deficiencies (Kwapata and Maliro 1999). Two of the most widely grown and consumed vegetables in Malawi are rape (*Brassica napus* L.) and Amaranthus—Bonongwe (*Amaranthus retroflexus* L*.)* (Gondwe 2008) and which therefore represent suitable targets for biofortification.

In temperate regions, *I* applied to soils is rapidly fixed into inert humus-bound forms and adsorbed onto Fe and Al hydrous oxides; these processes therefore restrict *I* availability to plants (Bowley et al. 2019; Shetaya et al. 2012). Following rainfall or fertiliser *I* input, only a narrow window of opportunity therefore exists for uptake by plant roots before *I* becomes unavailable. These processes have not yet been studied in tropical soils. This study therefore sought to investigate the potential for *I* biofortification of green leafy vegetables (*B. napus* and *A. retroflexus*) grown in tropical soils, using foliar and soil *I* application. The objective was to investigate plant *I* concentrations when grown in three contrasting tropical soils: a Vertisol, an Oxisol and an Alfisol, with soil or foliar *I* applications at levels of 0, 5 and 10 kg ha^−1^. Application rates were selected based on the previous work on green vegetables on other soil types, e.g. Lawson et al. (2015). Iodine distribution in the vegetables was also investigated for plants grown in the Alfisol with a single soil *I* application of 10 kg ha^−1^.

## Materials and methods

### Experimental design

Vertisol, Oxisol and Alfisol soils were collected from Ngabu in Chikwawa (15° 33′ S and 35° 11′ E), Bembeke in Dedza (14° 21′ S and 34° 35′ E) and Chitedze Research Station (13° 59′ S and 33° 35′ E), respectively. Soils were air-dried and sieved (< 4 mm) before being potted into 5 L pots (~ 6 kg pot^−1^ for Alfisols and Oxisols and ~ 5 kg pot^−1^ for Vertisols). A full set of 36 treatments, including all factorial combinations of three soils, two vegetable species, three *I* application rates and two methods of application for *I*, were examined in four randomised complete replicate blocks (144 pots in total), laid out in a glasshouse at Chitedze Research Station, Malawi.

Three seeds of either the exotic vegetable *B. napus* or the local vegetable *A. retroflexus* were sown in each pot. After four weeks, 3 g of NPKS fertiliser (0.69 g N; 0.63 g P; 0 g K; 0.12 g S) was applied to each pot as a basal dressing. Two weeks later, 3 g of calcium ammonium nitrate (CAN, 0.78 g N) was applied to each pot. These fertiliser treatments are equivalent to those stipulated in the Malawian Guide to Agriculture Production and Natural Resources Management (MoAFS 2015). Weeds were removed as soon as they appeared. Irrigation (c.100 mL pot^−1^ day^−1^) was applied by slowly pouring water directly onto the soil to avoid generation of leachate. Spider mites and other leaf pests were controlled by applying cypermethrin 200 ECE at a rate of 200 g L^−1^ as a general spray.

### Iodine application

Iodine solutions were prepared from sodium iodide (NaI) salt (AR grade, Fisher Scientific), and a single foliar or soil application was made to each pot four weeks after sowing. Soil applications consisted of 5 mL of solution added to the soil taking care to avoid contact with foliage; no other irrigation was provided that day to prevent leaching. Foliar *I* applications were applied using a calibrated spray set to discharge 1 mL of solution. Five mL was sprayed evenly over the leaves of each plant avoiding, as much as possible, loss to the soil.

### Harvesting

The first harvest of leaves was carried out 6 weeks after planting, 14 days after *I* application. Further harvests were scheduled at fortnightly intervals (28, 42, 56 and 70 days after *I* application). Leaves of *A. retroflexus* were collected by plucking the tips of the plant (c. 5 leaves) to simulate normal sequential harvesting. Similarly, *B. napus* leaves were plucked together with the mid-rib as normally consumed. Fresh weight (FW) was recorded before the leaves were carefully dry-brushed, washed with irrigation water and rinsed with deionised water, and was therefore broadly comparable with typical domestic cleaning of green vegetables before consumption. The leaves were then oven-dried in perforated paper bags at 40 °C for 48 h and the dry weight (DW) recorded. After the 5th harvest, the plant stems were removed, and the soil containing the plant roots was left to dry. The soil from each pot was then gently crushed using a pestle and mortar and sieved < 4 mm before being sub-sampled for analysis, re-potted and re-sown to assess the availability of residual *I*. This second crop was managed in an identical way to that of the first, including NPKS and CAN fertiliser applications.

### Iodine distribution within the vegetables

To investigate the distribution of *I* between leaves, stems and roots in the two vegetables, a separate randomised block experiment was conducted with fourfold replication in the Alfisol with a single soil *I* rate of 10 kg ha^−1^ applied 4 weeks after sowing. Other fertiliser treatments (NPKS and CAN) were applied, as previously outlined. Each plant was harvested 2 weeks after *I* application and separated into leaf, stem and roots. A subsequent crop of the same vegetable was immediately re-sown directly into each pot and allowed to grow for 6 weeks before it was also harvested and separated into its constituent parts. To avoid soil contamination, the harvested plants were thoroughly washed with irrigation water and then rinsed with deionised water prior to drying. Samples from both harvests were oven-dried at 40 °C for 48 h, weighed and then milled for analysis.

### Soil characterisation

Soil pH was measured following suspension in water (Milli-Q®, 18.2 MΩ cm; 10 g soil in 25 mL water) using a pH meter (Model pH 209, HANNA Instruments). The exchangeable cations; sodium (Na^+^), potassium (K^+^), magnesium (Mg^2+^), calcium (Ca^2+^) and aluminium (Al^3+^), were solubilised by extraction of 2 g air-dried soil (< 2 mm) in 20 mL of 1 M NH_4_NO_3_. The ‘effective cation exchange capacity’ (ECEC) was determined using the ‘Cohex’ method with cobalt (III) hexamine chloride solution using 25 mL of 0.0166 M Cohex and air-dried soil < 2 mm sieved soil (1.25 g of Vertisol and 2.5 g of either Alfisol or Oxisol; Ciesielski and Sterckeman 1997). Concentrations of soil hydrous oxides of iron (Fe), aluminium (Al) and manganese (Mn) were determined using the citrate bicarbonate dithionite (CBD) extraction method adapted from Kostka and Luther (1994) and Anschutz et al. (1998). All elemental analysis (Na, K, Mg, Ca, Co, Fe, Al, Mn) was carried out using inductively coupled plasma mass spectrometry (ICP-MS; Thermo–Fisher Scientific™ iCAP Q) operated in kinetic energy discrimination mode utilising He as a cell gas and Sc, Ge and Rh as internal standards to correct for drift. Soil organic carbon (%SOC) was determined using a Shimadzu TOC-VCPH analyser after acidification of the soil with HCl to liberate inorganic carbon. Organic carbon was then heated to 720 °C in the presence of a platinum-coated alumina catalyst and determined as CO_2_ using a non-dispersive infrared detector. Soil nitrogen (%N) was determined using a Leco TruMac CN analyser (Stockport, UK) after the soil was finely ground using a planetary ball mill (Retsch, PM 400).

### Iodine extraction

Soil *I* was extracted using tetramethyl ammonium hydroxide (TMAH, Sigma-Aldrich) using the method of Watts and Mitchell (2009). In brief, 10 mL of 10% TMAH was added to 4 g of air-dried, sieved soil (< 2 mm) before being heated at 70 °C for 3 h with gentle shaking after 1.5 h. The extracts were then centrifuged for 30 min at 3500 rpm and the supernatant filtered (0.22 µm) prior to analysis by ICP-MS. Plant *I* extraction used a modification of the method of Watts et al. (2010) employing microwave heating (MARS Xpress, CEM) to improve the extraction within a closed vessel system.

### Iodine analysis

Total *I* concentrations in soil and leaf vegetables was measured by ICP-MS using Rh and Re (10 μg L^−1^) as internal standards. Stock standards for *I* were prepared at 1000 mg L^−1^ of *I* from oven-dried KI and KIO_3_, and stored at 4 °C in 1% TMAH. Standards were freshly diluted in 1% TMAH or Milli-Q® water as required before each analytical run. Limits of detection (3 × standard deviation of operational blanks) were 0.047 µg L^−1^. Certified reference material BCR-129 (Hay) with a certified value for *I* of 0.167 mg kg^−1^ gave an average recovery of 92.8% and a standard error (*n* = 14) of 3.3%.

### Data analysis and presentation

Data were analysed with linear mixed models (LMM) using RStudio Desktop (1.2.1335) and the nlme library for the R platform (Pinheiro et al. 2017). In the LMM, the fixed effects were block and harvest batch, with the main effects and interactions of soil type, *I* application rate, *I* application method and vegetable type in factorial combination. The random effects were a between-pot and within-pot term, reflecting the repeated measurements on the same unit (pot) made in successive harvest batches. Two models were considered: the first (SP) assumed sphericity (i.e. a constant correlation between all within-pot effects) and the second (AR) assumed that the within-pot effect was an autoregressive random effect of order 1. These two models were compared on Akaike's information criterion (AIC). In all cases, the AR model was selected on this criterion and used for further inference.

The main effects of the factors with two or more degrees of freedom (*df*) were partitioned into prior 1-*df* contrasts as follows. The soil effect was partitioned into a contrast between Alfisol and Oxisol in one group and Vertisol (first contrast), and into the remaining orthogonal contrast between Alfisol and Oxisol. The former contrast is of interest because of the marked difference between the two groups of soils with respect to mineralogy, pH and base saturation (Lowole 1985) already known to have implications for soil geochemical processes (Cherian and Arnepalli, 2015).

The main effect of *I* rate (2 *df*) was partitioned with orthogonal polynomials to give a linear effect and a remainder. The interaction terms involving these two main effects were similarly partitioned into components involving these contrasts (e.g. the interaction of *I* rate and application method was partitioned into a component that corresponds to differences between the linear components of the *I* response under the two methods).

## Results and Discussions

### Soil characteristics

Soil characteristics are given in Table 1 and confirm that the soils selected had a range of contrasting properties. The sum of exchangeable cations, ECEC, soil organic carbon (SOC) and nitrogen (N) contents were all greatest in the Vertisol and least in the Oxisol. Total *I* concentration measured in the control soils, after the first crop, gave means of 4.13 mg kg^−1^ for the Oxisol, 5.88 mg kg^−1^ for the Alfisol and 5.66 mg kg^−1^ for the Vertisol.

**Table 1:** Initial chemical composition of Oxisol, Alfisol and Vertisol soils. Values are mean ± S.D. of 8 replicates for total* I* and 4 replicates for all other soil chemical properties

Soil	Total* I* (mg kg^−1^)	pH (water)	N (%)	SOC (%)	Co^111^ ECEC (cmol_C_ kg^−1^)	$\Sigma$ Ex. cations (cmol_C_ kg^−1^)	Al(OH)_3_ (g kg^−1^)	MnO_2_ (g kg^−1^)	Fe_2_O_2_ (g kg^−1^)
Oxisol	4.13 ± 0.50	4.20 ± 0.10	0.11 ± 0.01	2.01 ± 0.02	4.60 ± 0.20	3.10 ± 0.10	5.90 ± 0.60	0.48 ± 0.04	1.40 ± 0.10
Alfisol	5.88 ± 0.10	5.80 ± 0.20	0.14 ± 0.01	1.99 ± 0.03	11.0 ± 0.10	9.80 ± 0.50	3.00 ± 0.20	0.43 ± 0.04	0.77 ± 0.02
Vertisol	5.66 ± 0.20	8.40 ± 0.10	0.31 ± 0.03	2.65 ± 0.03	76.0 ± 1.00	77.0 ± 1.00	1.03 ± 0.01	1.00 ± 0.20	0.83 ± 0.02

### Dry biomass

The harvested total biomasses for *A. retroflexus* and *B. napus* are presented in Table 2a and b, respectively. The average yield differed significantly (*p* < 0.0001, EA Table 1) between soil types, vegetable types (4.90 and 4.14 g for *B. napus* and *A. retroflexus,*, respectively) and harvests, but was not affected by either *I* rate or *I* application method (*p* = 0.623, EA Table 1). In similar pot trials, Hong et al. (2008) reported that the addition of iodide as a soil amendment affected biomass production of four vegetables only at applied concentrations > 25 mg kg^−1^ which is c. 8 times the maximum concentration applied in this study, with phytotoxic effects occurring at 50 mg kg^−1^. Dai et al. (2006) observed no effect on spinach biomass for either iodide or iodate applications of up to 2 mg kg^−1^. Differences in yield as a function of soil type (Table 2) were clear, with greater biomass recorded for vegetables growing in the Vertisol where soil pH and nutrient status are greater than in Alfisols or Oxisols. No yield was recorded for some pots of the final harvest of the first crop, due to a red spider mite infestation towards the end of the experiment that had a greater effect on *A. retroflexus* than *B. napus.* Comparison of yield for the different *I* application levels suggested no phytotoxic effects on the growing vegetables, at 10 kg ha^−1^. This result is comparable to that of Lawson et al. (2015) who observed no phytotoxicity in field-grown Butterhead lettuce (*Lactuca sativa *L. var.* capitat*a) at *I* application levels of 7.5 kg ha^−1^, but saw chlorosis in plants subject to applications of 15 kg ha^−1^. The lower yield observed in the second crop compared to the first could be attributed to reduced soil nutrient pools despite fertiliser addition.

**Table 2:** Average dry biomass (g pot^−1^) for (a) *A. retroflexus* and (b) *B. napus* produced at different levels (0, 5 and 10 kg^−1^) of* I* application in the first and second crops grown up to 125 days after* I* application. Values are expressed as mean ± SE of four replicates

Harvest	Soil	Application method	Biomass (g pot^−1^) Crop 1				Biomass (g pot^−1^) Crop 2			
			0 kg ha^−1^* I*	5 kg ha^−1^* I*	10 kg ha^−1^* I*	Crop 1 mean	0 kg ha^−1^* I*	5 kg ha^−1^* I*	10 kg ha^−1^* I*	Crop 2 mean
*(a) Amaranthus retroflexus*										
1	Oxisol	Soil	3.14 ± 1.44	2.90 ± 2.38	4.49 ± 0.892	3.51	0.197 ± 0.035	0.165 ± 0.050	0.312 ± 0.107	0.24
		Foliar	2.82 ± 1.78	4.82 ± 2.43	1.75 ± 1.32	3.13	0.152 ± 0.067	0.370 ± 0.001	0.415 ± 0.152	0.29
	Alfisol	Soil	3.42 ± 0.72	5.73 ± 4.33	5.75 ± 2.25	4.97	0.415 ± 0.197	0.255 ± 0.115	0.237 ± 0.152	0.30
		Foliar	4.01 ± 3.17	4.09 ± 1.25	5.79 ± 4.51	4.63	0.720 ± 0.616	0.443 ± 0.277	0.435 ± 0.276	0.53
	Vertisol	Soil	4.51 ± 3.17	10.7 ± 5.65	10.8 ± 2.62	8.66	0.610 ± 0.264	0.613 ± 0.707	0.338 ± 0.152	0.52
		Foliar	6.98 ± 1.40	7.92 ± 3.30	7.76 ± 2.16	7.55	0.388 ± 0.265	0.487 ± 0.378	0.677 ± 0.434	0.52
2	Oxisol	Soil	6.48 ± 1.84	3.25 ± 1.45	3.83 ± 1.69	4.58	0.110 ± 0.096	0.292 ± 0.234	0.535 ± 0.350	0.33
		Foliar	4.83 ± 1.32	4.16 ± 1.17	3.21 ± 1.48	4.15	0.155 ± 0.088	0.970 ± 1.115	1.12 ± 0.593	0.73
	Alfisol	Soil	6.11 ± 2.20	6.21 ± 1.81	7.26 ± 0.001	6.28	0.548 ± 0.310	0.473 ± 0.416	0.522 ± 0.428	0.51
		Foliar	6.66 ± 1.20	5.65 ± 2.75	5.85 ± 1.00	6.05	1.26 ± 0.790	1.167 ± 0.531	0.512 ± 0.178	0.98
	Vertisol	Soil	11.1 ± 4.67	9.40 ± 2.06	11.7 ± 1.49	10.7	3.53 ± 1.65	2.68 ± 1.779	2.30 ± 0.867	2.85
		Foliar	10.6 ± 2.04	12.1 ± 2.16	10.4 ± 2.52	11.0	3.25 ± 0.992	2.12 ± 1.40	2.92 ± 1.161	2.76
3	Oxisol	Soil	1.10 ± 1.33	0.307 ± 0.021	0.253 ± 0.001	0.75	0.550 ± 0.462	1.50 ± 0.735	0.752 ± 0.420	0.82
		Foliar	1.34 ± 1.03	1.33 ± 0.563	1.29 ± 1.25	1.33	0.495 ± 0.120	0.380 ± 0.396	1.16 ± 0.687	0.80
	Alfisol	Soil	1.82 ± 1.60	1.85 ± 0.804	1.15 ± 0.667	1.59	0.677 ± 0.257	0.987 ± 0.391	0.502 ± 0.336	0.70
		Foliar	1.62 ± 2.22	1.90 ± 1.31	1.52 ± 0.915	1.68	1.41 ± 0.236	1.02 ± 0.405	0.980 ± 0.708	1.14
	Vertisol	Soil	2.81 ± 1.96	3.89 ± 2.14	5.73 ± 2.38	4.30	6.11 ± 2.08	4.68 ± 1.60	4.19 ± 1.87	4.99
		Foliar	3.70 ± 1.62	2.01 ± 0.288	4.31 ± 1.17	3.46	5.59 ± 1.59	4.27 ± 2.03	5.27 ± 1.87	5.04
4	Oxisol	Soil	0.502 ± 0.062	0.780 ± 0.354	0.365 ± 0.001	0.62	0.160 ± 0.098	0.370 ± 0.359	0.425 ± 0.214	0.31
		Foliar	0.119 ± 0.001	0.462 ± 0.343	0.088 ± 0.001	0.32	0.390 ± 0.428	0.250 ± 0.001	1.03 ± 0.514	0.69
	Alfisol	Soil	0.173 ± 0.187	0.339 ± 0.196	0.567 ± 0.775	0.38	0.357 ± 0.225	0.397 ± 0.219	0.330 ± 0.290	0.36
		Foliar	0	0.985 ± 0.477	0.140 ± 0.169	0.56	0.435 ± 0.331	0.723 ± 0.500	0.258 ± 0.035	0.45
	Vertisol	Soil	1.39 ± 1.99	1.27 ± 0.833	4.22 ± 0.468	2.49	3.19 ± 1.87	1.945 ± 1.36	3.31 ± 0.619	2.82
		Foliar	0.682 ± 0.335	0.400 ± 0.250	1.43 ± 0.470	0.95	2.82 ± 1.82	2.01 ± 0.779	2.72 ± 1.60	2.52
5	Oxisol	Soil	0.221 ± 0.001	1.79 ± 2.35	0	1.27	0.110 ± 0.018	0.515 ± 0.021	0.273 ± 0.145	0.25
		Foliar	0	0.036 ± 0.001	0	0.04	0.290 ± 0.255	0.130 ± 0.141	0.917 ± 0.669	0.56
	Alfisol	Soil	0	0	0.225 ± 0.125	0.23	0.220 ± 0.184	0.210 ± 0.044	0.545 ± 0.573	0.31
		Foliar	0	0.567 ± 0.001	0	0.57	0.420 ± 0.272	0.563 ± 0.119	0.763 ± 0.739	0.57
	Vertisol	Soil	1.39 ± 1.31	1.81 ± 0.288	2.66 ± 0.522	2.05	2.79 ± 1.38	2.00 ± 0.770	2.05 ± 1.03	2.28
		Foliar	1.04 ± 0.575	0.396 ± 0.001	1.45 ± 0.626	1.06	3.06 ± 0.935	1.64 ± 087	2.59 ± 0.667	2.43
*(b) Brassica napus*										
1	Oxisol	Soil	3.61 ± 2.10	0.97 ± 0.791	1.96 ± 0.746	2.18	0.517 ± 0.357	0.440 ± 0.189	0.527 ± 0.204	0.49
		Foliar	2.48 ± 0.300	3.12 ± 1.43	2.58 ± 0.505	2.73	0.397 ± 0.265	0.525 ± 0.279	0.215 ± 0.071	0.38
	Alfisol	Soil	2.92 ± 0.36	4.77 ± 1.75	5.20 ± 2.75	4.30	1.36 ± 0.096	1.36 ± 0.528	1.42 ± 0.645	1.38
		Foliar	3.76 ± 1.05	3.27 ± 1.02	5.17 ± 2.25	4.07	1.32 ± 0.703	0.752 ± 0.490	1.07 ± 0.065	1.04
	Vertisol	Soil	9.84 ± 1.47	11.5 ± 3.02	13.1 ± 1.58	11.5	1.32 ± 0.465	1.26 ± 0.654	1.29 ± 0.484	1.29
		Foliar	10.7 ± 1.53	13.0 ± 1.58	10.9 ± 2.72	11.5	1.31 ± 0.599	1.26 ± 0.303	1.59 ± 0.836	1.38
2	Oxisol	Soil	6.95 ± 2.50	4.28 ± 0.891	4.49 ± 1.23	5.24	0.387 ± 0.347	0.425 ± 0.269	0.540 ± 0.694	0.44
		Foliar	5.28 ± 1.59	4.48 ± 0.939	5.37 ± 2.01	5.04	0.900 ± 0.933	0.492 ± 0.367	0.318 ± 0.203	0.50
	Alfisol	Soil	5.47 ± 14.9	7.5 ± 2.71	9.09 ± 0.716	7.35	1.92 ± 1.02	1.36 ± 1.792	1.61 ± 0.602	1.63
		Foliar	6.61 ± 2.15	7.99 ± 1.10	9.05 ± 4.43	7.88	0.510 ± 0.238	0.623 ± 0.428	1.35 ± 0.731	0.83
	Vertisol	Soil	12.6 ± 6.17	15.2 ± 2.85	18.8 ± 2.54	15.5	4.41 ± 0.622	3.97 ± 2.18	3.89 ± 0.972	4.09
		Foliar	17.1 ± 3.68	19.1 ± 3.58	14.8 ± 2.60	17.0	4.60 ± 2.34	3.58 ± 1.29	5.32 ± 1.38	4.50
3	Oxisol	Soil	1.14 ± 0.08	1.503 ± 1.69	0.898 ± 0.418	1.18	0.850 ± 0.255	0.305 ± 0.064	1.77 ± 0.001	0.82
		Foliar	1.73 ± 0.68	1.73 ± 0.418	3.32 ± 2.34	2.21	1.87 ± 0.001	0.863 ± 0.673	0.290 ± 0.256	0.84
	Alfisol	Soil	2.42 ± 1.79	1.74 ± 0.530	1.83 ± 0.075	2.06	1.78 ± 0.746	1.90 ± 1.05	1.30 ± 1.41	1.61
		Foliar	1.88 ± 1.46	1.79 ± 1.26	2.81 ± 2.27	2.18	0.973 ± 0.671	0.703 ± 0.725	0.938 ± 0.715	0.88
	Vertisol	Soil	7.21 ± 1.67	6.97 ± 1.18	6.59 ± 1.37	6.93	9.07 ± 2.75	7.92 ± 1.96	7.95 ± 2.73	8.31
		Foliar	6.60 ± 1.34	7.43 ± 2.05	7.60 ± 1.41	7.21	8.70 ± 3.32	6.64 ± 1.084	7.69 ± 0.664	7.68
4	Oxisol	Soil	1.05 ± 0.646	1.07 ± 1.03	0.591 ± 0,306	0.87	0.587 ± 0.487	0.505 ± 0.163	1.34 ± 0.001	0.69
		Foliar	0.60 ± 0.391	1.34 ± 0.540	0.965 ± 0.803	0.97	1.15 ± 0.001	0.557 ± 0.576	0.205 ± 0.078	0.54
	Alfisol	Soil	1.07 ± 0.689	1.02 ± 0.306	1.62 ± 1.17	1.22	1.64 ± 0.951	0.690 ± 0.396	1.22 ± 1.75	1.28
		Foliar	1.62 ± 0.517	0.721 ± 0.599	1.50 ± 0.644	1.28	0.225 ± 0.050	0.490 ± 0.509	1.22 ± 1.38	0.73
	Vertisol	Soil	4.34 ± 0.646	2.32 ± 1.07	4.09 ± 0.883	3.58	3.11 ± 1.62	3.79 ± 1.77	2.59 ± 1.42	3.16
		Foliar	3.47 ± 1.07	4.23 ± 0.77	3.68 ± 1.10	3.79	4.32 ± 2.83	2.66 ± 1.412	3.06 ± 1.00	3.34
5	Oxisol	Soil	1.41 ± 1.37	0.378 ± 0.350	0.412 ± 0.328	0.80	0.910 ± 0.386	0.870 ± 0.976	1.77 ± 0.001	1.04
		Foliar	1.18 ± 0.917	1.39 ± 1.13	1.19 ± 0.884	1.26	1.49 ± 0.001	0.320 ± 0.198	0.555 ± 0.361	0.65
	Alfisol	Soil	1.58 ± 0.274	1.50 ± 0.147	1.45 ± 1.07	1.51	0.845 ± 0.657	0.500 ± 0.001	0.420 ± 0.589	0.64
		Foliar	1.40 ± 0.511	1.08 ± 1.15	1.23 ± 0.652	1.22	0.165 ± 0.064	0.387 ± 0.532	0.550 ± 0.464	0.39
	Vertisol	Soil	1.94 ± 0.968	2.29 ± 1.27	3.60 ± 0.599	2.61	3.58 ± 2.086	3.90 ± 1.113	3.02 ± 1.24	3.50
		Foliar	1.85 ± 0.887	2.79 ± 0.555	3.05 ± 0.726	2.50	4.36 ± 3.51	3.37 ± 1.99	3.03 ± 2.55	3.59

### Iodine contributions from irrigation water

The *I* concentration in the irrigation water used was 9.02 µg L^−1^ which contributed *I* to the plants from daily watering. A total of 88 µg pot^−1^ was added to the irrigated soil over five harvests (Table 3), which is small in comparison with the total soil *I* in each pot that was in the range 4130–5880 µg pot^−1^ (Table 1). However, *I* in irrigation water is much more available to plants than soil *I* (Bowley et al. 2019).

**Table 3:** Cumulative iodine contribution from irrigation water across five harvests (crop 1)

Irrigation iodine	Irrigation addition	Iodine addition	Harvest 1 41 days	Harvest 2 55 days	Harvest 3 69 days	Harvest 4 83 days	Harvest 5 97 days
(µg L^−1^)	(L d^−1^ pot^−1^)	(µg pot^−1^ d^−1^)	(µg pot^−1^)	(µg pot^−1^)	(µg pot^−1^)	(µg pot^−1^)	(µg pot^−1^)
9.02	0.1	0.902	37.0	49.6	62.2	74.9	87.5

**Fig. 1:**
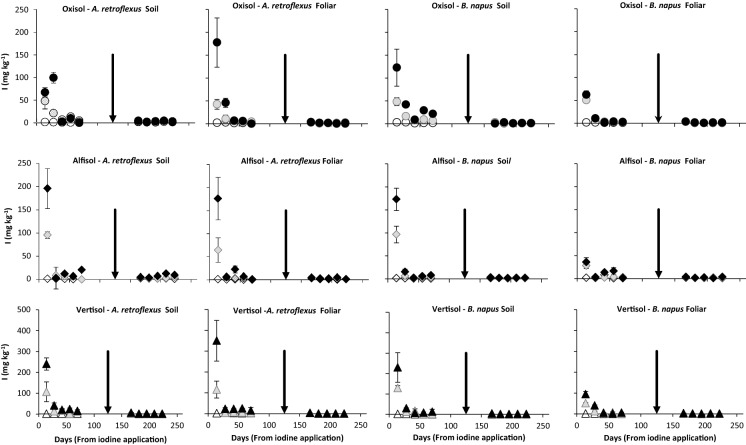
Iodine concentration (*I*_C_ mg kg^−1^) in *A. retroflexus* and *B. napus* grown on Oxisol, Alfisol and Vertisol soils and subject to repeat harvests at 14-day intervals after foliar or soil *I* application at levels of 0, 5 and 10 kg ha^−1^ *I*. The arrow indicates timing of sowing of a second crop. Key: open symbol = 0 kg ha^−1^, grey symbol = 5 kg ha^−1^, black symbol = 10 kg ha^−1^. Note the different Y-axis scale for Vertisol data to accommodate higher values. Error bars represent the standard error of the mean (SEM) for 4 replicates

Average leaf *I* concentrations of 1.69 mg kg^−1^ DW and 1.00 mg kg^−1^ DW were recorded for *A. retroflexus* and *B. napus*, respectively, grown as controls (Fig. 1). These concentrations are tenfold greater than observed by Watts et al. (2015) for field-grown vegetables in Malawi that were collected from non-calcareous (0.07 mg kg^−1^ *A. retroflexus*, 0.05 mg kg^−1^ *B. napus)* and calcareous soils (0.16 mg kg^−1^ *A. retroflexus*, 0.15 mg kg^−1^ *B. napus*). This may indicate that the irrigation water was a significant source of *I* to the control plants, as also found by Bowley et al. (2019) for an ^129^*I*-labelled pot experiment. Assuming an adult consumed 30 g DW d^−1^ (Joy et al. 2015) of the control vegetables grown in this study, they would ingest between 0.005 and 0.007 mg day^−1^ of *I* compared to a daily requirement of 0.15 mg (WHO 2007). Rainfall *I* concentrations, reported to be in the range 0.5–5 µg L^−1^ (Neal et al. 2007; Hou et al. 2009), would be very unlikely to increase the *I* concentration in green vegetables sufficiently to attain the recommended daily *I* intake and rainfall would not provide the consistent daily input from the irrigation water in this experiment. To achieve the recommended dietary allowance (RDA) for *I* from biofortification of both irrigated and rain-fed crops is a plausible approach (Signore et al. 2018; Dai et al. 2006; Zhu et al. 2004; Voogt et al. 2010), especially for communities where availability, quality or use of iodised salt is problematic.

### Plant *I* concentrations (*I*_C_) and uptake (*I*_U_)

**Fig. 2:**
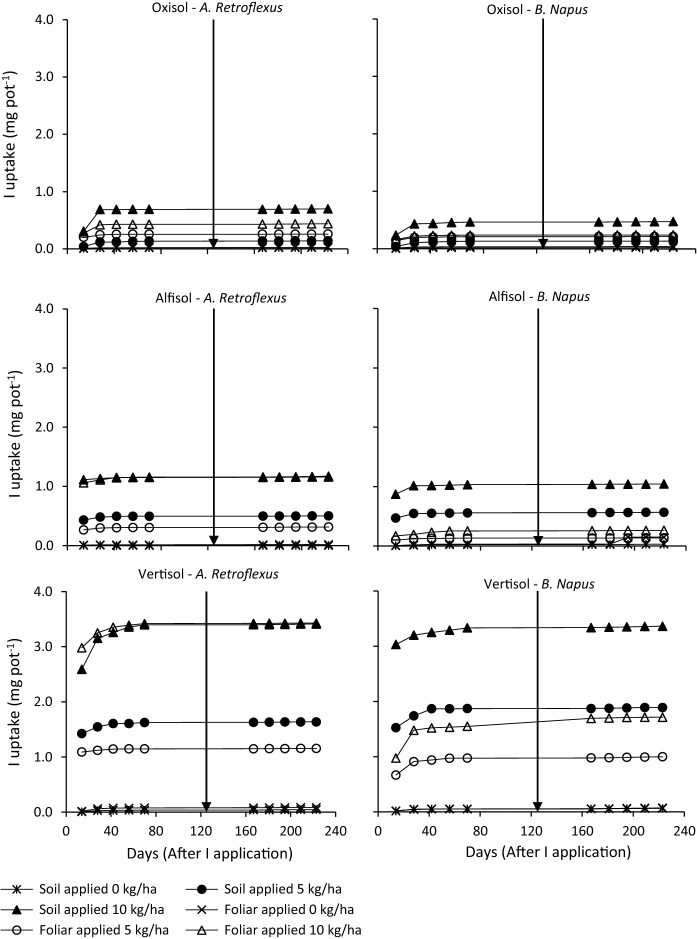
Comparison of cumulative *I*_U_ (mg pot^−1^) in repeated harvests of *A. retroflexus* and *B. napus* grown on Oxisol, Alfisol and Vertisol soils with 0, 5 and 10 kg ha^−1^ soil or foliar *I* application. Samples were harvested at 14-day intervals. The arrow indicates planting of a second crop

Plant *I* concentrations (*I*_C_) are shown in Fig. 1 and cumulative *I* uptake (*I*_U_) in Fig. 2. Significant differences were observed between soil type in terms of *I*_C_ and *I*_U_, p = 0.003 and *p* < 0.001, respectively (EA Table A). However, the contrasts between soil types in EA Table B show no differences (*p* = 0.354) in *I*_C_ between the Alfisol and Oxisol, considered as a group, versus Vertisol. By contrast, there is strong evidence for a difference in *I*_U_ (*p* < 0.0001) as more *I* was taken up by plants growing in the Vertisols compared to the mean uptake for those growing in Alfisol and Oxisols. For example, 14 days after soil *I* application of 10 kg ha^−1^, crop *I*_U_ was 2.59 and 3.03 mg pot^−1^ in the Vertisol, and 0.710 and 0.556 mg pot^−1^ in the Alfisol and Oxisol, for *A. retroflexus* and *B. napus*, respectively. For foliar application, *I*_U_ was 2.96 and 0.977 mg pot^−1^ in *A. retroflexus* and *B. napus* grown in the Vertisol; equivalent values of *I*_U_ for the Alfisol and Oxisol combined were 0.669 and 0.165 mg pot^−1^. Whilst *I*_C_ in plants grown on the Alfisol were significantly greater than those grown on the Oxisol (p = 0.001), there was no significant difference in *I*_U_ (*p* = 0.627) between these acidic soils (EA Table B). At low soil pH, Al toxicity can affect root development (Panda et al. 2009), thereby retarding nutrient/iodine uptake from soil and compromising aboveground vegetative growth, thereby reducing the leaf area available for foliar *I* application.

There was strong evidence for a difference in *I*_C_ between the two green vegetables *A. retroflexus* (36.8 mg kg^−1^) and *B. napus* (20.8 mg kg^−1^) (EA Table A, *p* < 0.0001); however, there was no evidence for a corresponding difference in *I*_U_ (EA Table A, *p* = 0.897). This may suggest differences in the uptake efficiency of the applied *I* between these vegetables. However, in this study the *I*_U_ is affected by differences in biomass production for these vegetables which was in the sequence of Vertisol > Alfisol > Oxisol (Table 2). The *I*_C_ data confirm the findings of Dai et al. (2004) and Gonnella et al. (2019) that green vegetable species show variations in *I*_C_ and efficacy of translocation within the plant.

In all cases, initial plant *I*_C_ was greatest when receiving the most *I* and declined with decreasing application rate, in the sequence 10 kg ha^−1^ (55.2 mg kg^−1^) > 5 kg ha^−1^ (24.7 mg kg^−1^) > 0 g ha^−1^ (1.53 mg kg^−1^) (Fig. 1). Corresponding average *I*_U_ values were 0.392, 0.186 and 0.009 mg pot^−1^ at 10, 5 and 0 kg ha^−1^, respectively (Fig. 2). EA Table B shows strong evidence for both linear and nonlinear effects of *I* application rate (*p* < 0.0001) on plant *I*_C_ and *I*_U_, respectively. Similarly, Dai et al. (2004) reported a significant increase in both *I*_C_ in the edible parts of vegetables and in soil-to-plant transfer factors with increasing *I* application.

Regardless of application rate, there was strong evidence for a greater *I*_C_ using soil, compared to foliar, application, with means of 30.8 and 22.9 mg kg^−1^, respectively (EA Table A and EA Table B, *p* = 0.006), but much weaker evidence for a corresponding difference in *I*_U_ (p = 0.06, EA Table A and EA Table B). Again, this reflects differences in vegetable biomass (Table 2) with significant variations observed with harvest, soil type and vegetable species (*p* < 0.0001, EA Table A).

There was a significant decline in both *I*_C_ and *I*_U_ with harvest number, *p* < 0.0001 (EA Tables A & B). A significant decrease in *I*_C_ and *I*_U_ in both crops, for both soil and foliar applications at 5 and 10 kg ha^−1^, was observed between the first and second harvest; decreases in *I*_C_ of > 60% for *A. retroflexus* and > 80% for *B. napus* were observed between harvest 1 and 2. Both *I*_C_ and *I*_U_ continued to decrease with subsequent harvests and by harvest 3 these were indistinguishable from the control plants (Figs. 1 and 2). This suggests that the *I* applied was available in the soil for a relatively short period of time before becoming fixed. Soil *I* retention and bioavailability are strongly influenced by factors affecting adsorption strength, including soil pH, redox, soil organic matter (OM), oxides of iron (Fe) and aluminium (Al), clay content and mineralogy (Shetaya et al., 2012). Adsorption of *I* was also shown by Dai et al. (2004) to be positively correlated with the concentrations of iron and aluminium oxides in 20 Chinese soils. It is likely that *I* applied to an acidic Oxisol with a pH of 4.2, and high concentrations of Al(OH)_3_, MnO_2_ and Fe_2_O_2_ oxides (Table 1), would be rapidly sorbed into soil oxides and humus and therefore rendered unavailable for plant uptake. There is also the possibility that soil-applied *I* could be lost through volatilisation (Amachi et al. 2003; Muramatsu et al. 1989) although the study of Shetaya et al. (2012) indicates that this is typically very small.

There was a negligible change in *I*_C_, and cumulative *I*_U_, after harvest 2 for plants subject to foliar *I* treatment (Figs. 1 and 2). This simply reflects the absence of replenishment from soil *I* applications. Iodine in subsequent harvests is likely to be derived from either *I* on the plant stem that translocated after spraying, or spray that had drifted onto newly growing shoots present at the time of spraying, but too young to be picked at the first harvest. This highlights that for foliar application sufficient spreading of *I* on the edible leaves is crucial in order to attain effective biofortification (Lawson et al. 2015). Therefore, regardless of application method (soil or foliage), the results suggest that green vegetables subjected to repeated harvests would require repeated *I* applications between harvests in order to optimise *I*_C_ in the newly sprouting leaves. An alternative might be continued supply of *I* in irrigation water throughout the productive lifetime of the plant.

Leaf *I*_C_ and *I*_U_ in the second sowing of both vegetables was negligible, with an *I*_C_ range of 0.001 to 3.0 mg kg^−1^ and average cumulative uptakes < 0.0065 mg pot^−1^ (Figs. 1 and 2). Thus, very little residual labile *I* was available to the second crop. Values of *I*_C_ and *I*_U_ for the second crop were almost indistinguishable from the controls suggesting that the main source of *I* to the second crop was irrigation water.

### The potential contribution of fertiliser derived *I* to dietary *I* supply

Sample preparation included washing of the vegetables with irrigation water followed by rinsing with deionised water and was therefore broadly comparable with typical domestic cleaning of green vegetables before consumption. The potential contributions to dietary *I* supply from consumption of 30 g DW day^−1^ of a plant harvested from the Vertisol 14 days after a 10 kg ha^−1^ soil *I* application were 0.94 and 0.97 mg day^−1^ for *A. retroflexus* and *B. napus*, respectively; equivalent values for the Oxisol were 0.26 and 0.52 mg day^−1^. Foliar application values were 1.37 or 0.69 mg day^−1^ from *A. retroflexus* and 0.40 or 0.26 mg day^−1^ for *B. napus,* harvested 14 days after a 10 kg ha^−1^ *I* application to a Vertisol and Oxisol, respectively. Considering an RDA for *I* of 0.15 mg day^−1^, results suggest that both soil and foliar *I* application to green vegetables growing in tropical soils can therefore increase dietary *I* intake. However, the time period over which an increase in *I*_C_ is observed in the plant is limited by either *I* fixation in the soil or by repeated harvests of treated leaves. Foliar application of *I* appeared to have some advantage over soil application when plants were growing in acidic soils where *I* fixation was likely to be most rapid. An approach to mitigate this effect would be to establish whether liming of acidic soils when using soil, *I* application reduces the rate of *I* fixation, as well as being likely to reduce Al toxicity effects. Nevertheless, the results from vegetables growing on the Vertisol (pH 8.4) suggest that continuous inter-harvest *I* applications would still be necessary to maintain the viability of *I* biofortification of green vegetables.

### Residual soil iodine concentrations

**Fig. 3:**
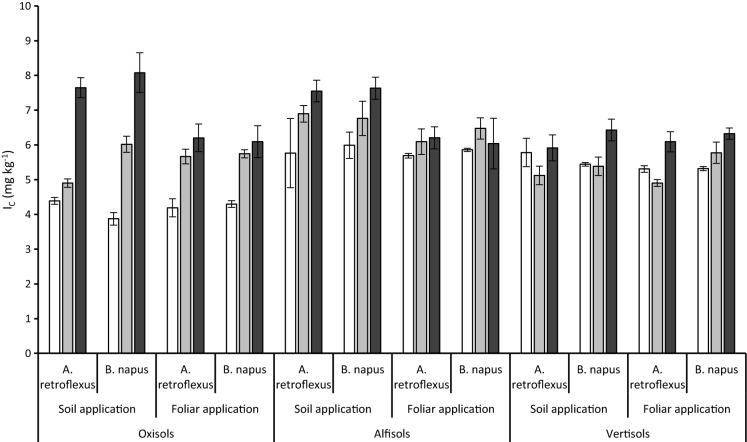
Soil *I*_C_ (mg kg^−1^) after the first crop (5th harvest), 125 days after soil *I* application. Key: open bars = 0 kg ha^−1^, grey bars = 5 kg ha^−1^, black bars = 10 kg ha^−1^. Error bars are the standard error of the mean of 4 replicates

Soil *I*_C_ measured after the fifth harvest of the first crop is shown in Fig. 3. Compared to control soils, the greatest increase in soil *I* was observed in Oxisols, with the smallest changes observed in Vertisols. In comparison with the control, 47, 28 and 11% of the applied *I* (either in irrigation or as a soil application) was retained in the Oxisol, Alfisol and Vertisol, respectively.

The retention of *I* in the Oxisols can be attributed to both the low plant biomass and consequent *I* uptake observed on these soils (Table 2), and fixation of the *I* in the soil. In the Vertisols, the small increase in soil *I* implies that more of the applied *I* was available to the growing plants which is confirmed by the observed higher *I*_U_ (Table 2 and Fig. 2) despite much greater yields.

### Iodine distribution within the vegetables

**Fig. 4:**
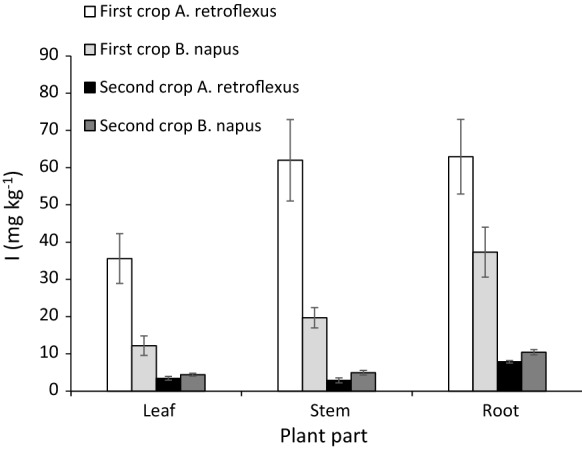
Iodine concentration (mg kg^−1^) in the leaves, stems and roots of *A. retroflexus* and *B. napus* grown in an Alfisol*.* The first crop was harvested 2 weeks after soil *I* application (10 kg ha^−1^); the second crop was sown immediately after harvesting the first and was harvested 6 weeks later. Error bars are the standard error of the mean of 4 replicates

The distribution of *I* within plants grown in the Alfisol with an *I* application of 10 kg ha^−1^ to the soil is shown in Fig. 4. In plants harvested two weeks after *I* application, the roots and stems of both vegetables had greater *I*_C_ than the leaves. This pattern was also seen in a second crop harvested six weeks later (Fig. 4); however, *I*_C_ was also lower by 90% for *A. retroflexus* and 64% for *B. napus* compared to the first crop. This observation agrees with previous studies in which *I* concentrations in plant tissues decreased in the order root > stem > leaf (e.g. Weng et al. 2013; Hong et al. 2008; Mackowiak and Grossl 1999). Greater *I*_C_ in the roots and stems indicates that *I* can be accumulated within these parts with only a small proportion transported to the leaves. This raises the possibility that where plants are subjected to repeated harvesting *I* present in leaves in subsequent harvests may originate from the roots and stem after initial soil uptake. Also, stores of *I* in roots and stems ploughed into the soil, as residues or as green manure, may provide *I* to subsequent crops.

## Conclusions

Soil and foliar *I* biofortification to green vegetables substantially increased *I*_U_ by plants and has the potential to increase dietary *I* supply. However, soil and foliar *I* biofortification to green vegetables that are subjected to repeated harvests would require repeated biofortification to consistently enhance *I*_C_ in the harvested leaves. Soil acidity control, measures such as input of liming materials, should be considered in acidic soils in order to enhance soil *I*_U_ and optimise leaf biomass production in green vegetables. Further investigations are required to determine (i) the cost-effectiveness of *I* biofortification of green vegetables, (ii) whether soil- and foliar-applied *I* is retained in cooked vegetables (using locally relevant recipes) and (iii) the extent to which leaf *I* is bioavailable to consumers.

## Electronic supplementary material

Below is the link to the electronic supplementary material.

Supplementary file1 (DOCX 27 kb)
