# Supplementary material for: Agronomic iodine biofortification of leafy vegetables grown in Vertisols, Oxisols and Alfisols
Source: Environ Geochem Health. 2020 Sep 23;43(1):361–74. doi: 10.1007/s10653-020-00714-z (PMC7847871; doi:10.1007/s10653-020-00714-z)
Supplement: Supplementary file 1 — Supplementary file1 (DOCX 27 kb) [file 10653_2020_714_MOESM1_ESM.docx]

**Electronic Appendix**

**EA Table A**: Analysis of variance for plant iodine concentration (I_C_), cumulative iodine uptake (I_U_) and biomass weight in crop 1.

|  | **Degrees of freedom** | | |  | **P-values** | | |
| --- | --- | --- | --- | --- | --- | --- | --- |
|  | **Numerator** | | **Denominator** |  | **I_C_** | **I_U_** | **Biomass** |
| Block | | 3 | 123 |  | 0.0234 | 0.0480 | 0.567 |
| Harvest | | 4 | 440 |  | <0.0001 | <0.0001 | <.0001 |
| Soil | | 2 | 123 |  | 0.0029 | <0.0001 | <.0001 |
| GV | | 1 | 123 |  | <0.0001 | 0.8972 | <.0001 |
| I | | 2 | 123 |  | <0.0001 | <0.0001 | 0.379 |
| Method | | 1 | 440 |  | 0.0062 | 0.0604 | 0.623 |
| Soil x GV | | 2 | 123 |  | <0.0001 | 0.0136 | 0.069 |
| Soil x I | | 4 | 123 |  | 0.3049 | 0.0118 | 0.028 |
| GV x I | | 2 | 124 |  | 0.0747 | 0.0946 | 0.409 |
| Soil x Method | | 2 | 440 |  | 0.0534 | 0.3734 | 0.221 |
| GV x Method | | 1 | 440 |  | 0.0109 | 0.2933 | 0.202 |
| I x Method | | 2 | 440 |  | 0.1756 | 0.2145 | 0.713 |
| Soil x GV x I | | 4 | 123 |  | 0.1487 | 0.240 | 0.124 |
| Soil x GV x Method | | 2 | 440 |  | 0.5948 | 0.8133 | 0.033 |
| Soil x I x Method | | 4 | 440 |  | 0.3739 | 0.0635 | 0.187 |
| GV x I x Method | | 2 | 440 |  | 0.0945 | 0.2344 | 0.057 |
| Soil x GV x I x Method | | 4 | 440 |  | 0.2641 | 0.3728 | 0.239 |

GV = Green vegetable; I = iodine application level

**EA Table B:** ANOVA table of contrasts for I concentration (I_C_) and cumulative I uptake (I_U_).

|  | **Degrees of freedom** | |  | **P-values** | |
| --- | --- | --- | --- | --- | --- |
| **Contrasts** | **Numerator** | **Denominator** |  | **I_C_** | **I_U_** |
| Block | 3 | 123 |  | 0.0234 | 0.0481 |
| Harvest | 4 | 440 |  | <.0001 | <.0001 |
| Alfox vs Vert | 1 | 123 |  | 0.3538 | <.0001 |
| Alf vs Ox | 1 | 123 |  | 0.001 | 0.6265 |
| GV | 1 | 123 |  | <.0001 | 0.8972 |
| I_lin | 1 | 123 |  | <.0001 | <.0001 |
| I rem | 1 | 123 |  | <.0001 | <.0001 |
| Method | 1 | 440 |  | 0.0062 | 0.0604 |
| GV x Alfox x Vert | 1 | 123 |  | <.0001 | 0.0034 |
| GV x Alf x Ox | 1 | 123 |  | 0.3351 | 0.9896 |
| I lin x Alfox x Vert | 1 | 123 |  | 0.3445 | 0.0077 |
| I rem x Alfox x Vert | 1 | 123 |  | 0.2733 | 0.2919 |
| I lin x Alf x Ox | 1 | 123 |  | 0.1217 | 0.0353 |
| I rem x Alf x Ox | 1 | 440 |  | 0.5558 | 0.4835 |
| Alfox x Vert x Method | 1 | 440 |  | 0.0266 | 0.169 |
| Alf x Ox x Method | 1 | 440 |  | 0.3872 | 0.9899 |
| GV x I lin | 1 | 123 |  | 0.0417 | 0.0291 |
| GV x I rem | 1 | 123 |  | 0.2629 | 0.912 |
| GV x method | 1 | 440 |  | 0.0109 | 0.2933 |
| Method x I_lin | 1 | 440 |  | 0.0742 | 0.091 |
| Method x I rem | 1 | 440 |  | 0.5901 | 0.638 |
| GV x Alfox x Vert x I lim | 1 | 123 |  | 0.0395 | 0.4852 |
| GV x Alfox x Vert x I rem | 1 | 123 |  | 0.1266 | 0.0316 |
| GV x Alf x Ox x I lim | 1 | 123 |  | 0.980 | 0.9202 |
| GV x Alf x Ox x I rem | 1 | 123 |  | 0.6518 | 0.5559 |
| Alfox x Vert x GV x Method | 1 | 440 |  | 0.8914 | 0.8378 |
| Alf x Ox x GV x Method | 1 | 440 |  | 0.3127 | 0.5425 |
| GV x I lin x Method | 1 | 440 |  | 0.2041 | 0.5744 |
| GV x I rem x Method | 1 | 440 |  | 0.0674 | 0.1038 |
| Alfox x Vert x I lin x Method | 1 | 440 |  | 0.6601 | 0.1988 |
| Alfox x Vert x I rem x Method | 1 | 440 |  | 0.2866 | 0.461 |
| Alf x Ox x I rem x Method | 1 | 440 |  | 0.2787 | 0.5813 |
| Alf x Ox x I lim x Method | 1 | 440 |  | 0.2198 | 0.0110 |

Alfox = alfisol plus oxisol, Alf = alfisol, Ox = oxisol, Vert = vertisol, GV = green vegetable, I lin = iodine level linear contrast and I rem = iodine level non-linear contrast.
